# Surface Modification of Polymers by Plasma Treatment for Appropriate Adhesion of Coatings

**DOI:** 10.3390/ma17071494

**Published:** 2024-03-26

**Authors:** Gregor Primc, Miran Mozetič

**Affiliations:** Department of Surface Engineering, Jozef Stefan Institute, Jamova cesta 39, 1000 Ljubljana, Slovenia; gregor.primc@ijs.si

**Keywords:** polymers, gaseous plasma, wettability, adhesion, coatings

## Abstract

In this study, recent advances in tailoring the surface properties of polymers for the optimization of the adhesion of various coatings by non-equilibrium gaseous plasma are reviewed, and important findings are stressed. Different authors have used various experimental setups and reported results that scatter significantly and are sometimes contradictory. The correlations between the processing parameters and the adhesion are drawn, and discrepancies are explained. Many authors have explained improved adhesion with the adjustment of the surface free energy or wettability of the polymer substrate and the surface tension of liquids used for the deposition of thin films. The adhesion force between the polymer substrate and the coating does not always follow the evolution of the surface wettability, which is explained by several effects, including the aging effects due to the hydrophobic recovery and the formation of an interlayer rich in loosely bonded low molecular weight fragments.

## 1. Introduction

**Figure 1:**
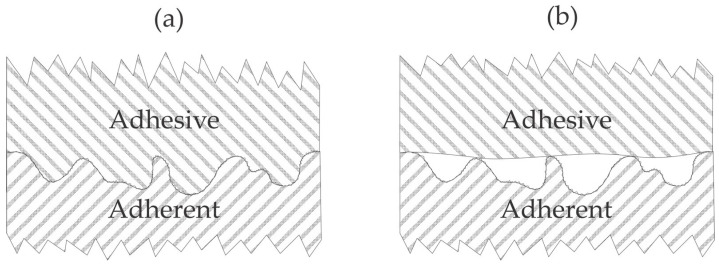
Influence of morphology on the optimal (**a**) and inadequate (**b**) adhesion between two materials.

The adhesion of various coatings on polymer substrates has attracted significant attention from the scientific community due to the need to invent reliable, inexpensive, and ecologically benign methods for optimizing the adhesion force and durability of products made from adhered materials. The interaction between the substrate and the coating has also been studied, and numerous mechanisms have been proposed [1]. Briefly, the mechanisms could be categorized as mechanical coupling and chemical interaction. The mechanical coupling will be enhanced for substrates of appropriate roughness, while the chemical interaction will be optimized if the deposited material forms covalent or other strong bonds with the functional groups on the polymer surface. A crucial property that affects both mechanical and chemical interaction is the ability of a coating to fill any gaps or pores on the surface of materials to be joined. The effect is illustrated in Figure 1.

Figure 1a illustrates the case of optimal adhesion. The materials stick well because all gaps or other morphological features are filled. The optimal adhesion is rarely achieved since the coating does not wet the substrate thoroughly, i.e., does not fill tiny pores or gaps. The inadequate adhesion is illustrated in Figure 1b. In this case, the interface between the two materials is minimal because the coating does not wet the substrate. This effect is often observed when the substrate is hydrophobic and rough on the sub-micrometer scale, and the coating is hydrophilic. The effect illustrated in Figure 1b could be suppressed by pressing the substrate and the coating together to force the filling of at least some gaps or pores. An alternative solution is the application of ionic liquids for coating various substances onto the polymer surfaces [2]. Yet another alternative is the hydrophilization of the substrate. Hydrophilization can be achieved by chemical methods, often by depositing an intermediate layer that balances the substrate’s surface free energy and the coating’s surface tension. These chemicals are usually various primers, which may form a very thin film on the substrate. An alternative to chemical methods for balancing the substrate’s surface properties and/or coating is the application of gaseous plasma. Gaseous plasma is a source of particles with considerable kinetic or potential energy and radiation, of which the photon energy often exceeds the binding energy of atoms in solid materials. Numerous authors have reported beneficial results when using gaseous plasma. Selected recent scientific articles are briefly reviewed, and the results are interpreted in the view of plasma science.

## 2. Gaseous Plasma

Gaseous plasma is often called the fourth state of matter, but it is actually gas with a significant concentration of free electrons and positively charged ions. Polymer materials are usually treated with non-equilibrium gaseous plasma, a state of the gas in which the temperature of different particles deviates significantly. The translational temperature of heavy particles (all plasma particles except electrons) is usually just above the room temperature, while the electron temperature is often of the order of 10,000 K. The rotational temperature of molecules is often close to the translational temperature, but the vibrational temperature could be several 1000 K. The degree of ionization and dissociation is much larger than calculated from the Boltzmann distribution, taking into account the translational temperature.

The electron temperature is usually much larger than the translational temperature of other gaseous particles, like molecules and ions [3]. The translational temperature of molecules in plasmas suitable for the treatment of polymer materials is often close to room temperature, and the electron temperature usually exceeds 10,000 K. Such a large electron temperature enables inelastic collisions with gaseous molecules, which leads to the excitation, dissociation, or ionization of the molecules [4]. The degrees of excitation, dissociation, and ionization in non-equilibrium gaseous plasma are much larger than predicted by the Boltzmann equation, taking into account the translational temperature of molecules. Some plasmas exhibit vibrational temperatures of several 1000 K, almost irrespective of the gas translational temperature [5]. The electronically excited molecules and atoms may be de-excited by radiation, usually in the vacuum ultraviolet range, i.e., with the photon energy between 6 and 12 eV [6]. Therefore, the major factors governing the interaction of non-equilibrium gaseous plasma with polymer materials will be the fluxes of plasma species on the polymer surface: positively charged ions, neutral radicals, including atoms in the ground state, molecules and radicals in metastable excited states, and photons in the UV or VUV range, and not the gas translational temperature or the temperature of the polymer surface. The kinetic energy of ions impinging on the polymer surface is also essential if the polymer surface is biased against the plasma.

The interaction of plasma species (charged particles, molecular radicals, and excited species) with solid surfaces is always exothermic. It often leads to the modification of the surface composition and structure, so it is suitable for tailoring surface properties of various materials [7]. Non-equilibrium plasmas are often used on an industrial scale to modify polymer wettability [8]. The exact mechanisms of plasma–polymer interaction are still inadequately understood, and recent theoretical articles have enlightened the phenomenon’s complexity [9,10]. In general, the plasma treatment causes at least one (more frequently several) of the following effects:

- Heating;
- Substitution of surface atoms with atoms from plasma (often called functionalization);
- Breaking polymer chains, depolymerization, and formation of polymer fragments;
- Etching;
- Modification of polymer morphology;
- Modification of the elasticity of the polymer surface film;
- Breaking bonds in the surface film and possible cross-linking;
- Modification of surface wettability.

The latter is particularly interesting when studying the adhesion of coatings on polymer materials. The wettability reflects the ability of the coating to penetrate gaps and pores (Figure 1). Suppose the surface is highly wettable (hydrophilic). In that case, the soft or melted materials, let alone liquids, will enter the pores because of the capillary forces, so the effect illustrated in Figure 1a is feasible. If the wettability is poor, the effect illustrated in Figure 1b will likely occur.

## 3. Review of Recent Literature

Numerous authors have reported using plasma treatment to increase the adhesion of various coatings on polymer materials. Only selected recent papers published since 2022 are here reviewed, and the observations are explained and illustrated.

### 3.1. High-Pressure Plasmas

A large frequency of collisions between gaseous particles governs the atmospheric pressure plasmas. The foundations of atmospheric pressure plasmas have been presented by Bruggeman et al. [11]. Briefly, the collision frequency (average number of collisions per unit of time) increases linearly with the increasing density of gaseous particles, which is proportional to the gas pressure and inversely proportional to the translational temperature of colliding particles. The collision frequency between molecules, atoms, and ions is roughly 1 MHz, and the electron-neutral collision frequency is as large as about 1 THz [11]. Such a large frequency of collisions causes intensive kinetic energy exchange between the colliding particles, so the electron temperature in atmospheric pressure non-equilibrium plasma is rarely above 1 eV. The kinetic energy of positively charged ions impinging on the surface of a plasma-treated material is often negligible, i.e., below 1 eV. Furthermore, the probability of three-body collisions increases as the square of the density of colliding species [12]. The loss of molecular radicals in the gas phase occurs predominantly by three-body collisions, so the lifetime of radicals such as atoms is as low as about 1 µs at atmospheric pressure. Thus, the molecule dissociation fraction is much lower at atmospheric pressure than in low-pressure plasmas, and a homogeneous plasma in a large volume is very difficult to achieve.

**Figure 2:**
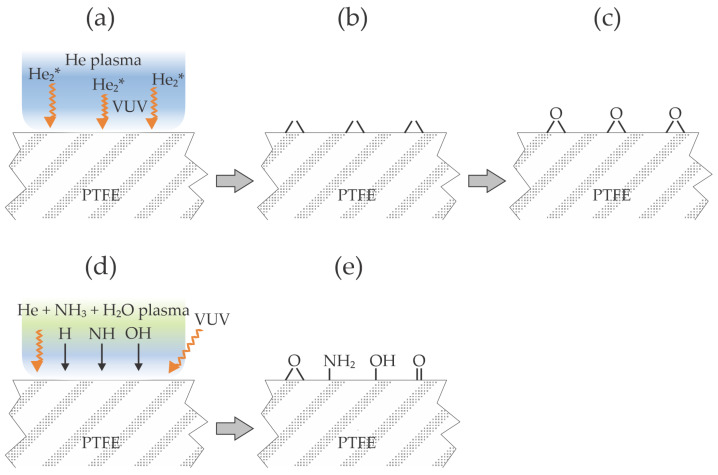
Illustration of the interaction between plasma sustained in pure helium (**a**–**c**) or a mixture of helium with ammonia water (**d**,**e**) with the surface of the PTFE.

Li et al. [13] treated polytetrafluoroethylene (PTFE) with helium plasma and added ammonia water to the processing gas. The optimal pressure in the processing chamber was 0.5 MPa, and the total gas flow rate was 4 L/min. The wettability was evaluated by measuring the static water contact angle (WCA), while the adhesion of the epoxy glue was evaluated by peel strength experiments. The plasma generator operated at the frequency of 60 kHz, with a maximal voltage of 5.12 kV. The ammonia water and helium mixing ratio varied between 0 and 2.5 vol.% of ammonia water. The plasma-treated samples were also characterized by X-ray photoelectron spectroscopy (XPS), and roughness was evaluated by atomic force microscopy (AFM). The PTFE treatment with pure helium plasma improved wettability because the WCA dropped from 110° to 40° after 15 s and 30° after about 120 s. After that, the WCA remained constant. Although the authors did not mention it, the increased wettability is attributed to the absorption of vacuum ultraviolet radiation, which causes structural changes in the PTFE surface film [14]. The addition of ammonia water caused a further decrease in the WCA to about 20°, and the optimal admixture was about 1 vol%. XPS results revealed the formation of oxygen-containing functional groups even on the sample treated with pure helium plasma, and the concentration of these groups increased significantly with a small admixture of ammonia water to the plasma gas. The authors even recognized some C–N bonds in de-convoluted high-resolution C1s spectra. AFM did not reveal noticeable morphological changes, but the pristine samples were relatively rough, so any changes might have been hidden. Namely, OH and O radicals (from water) always cause laterally non-homogeneous etching of the PTFE materials, as explained in [15]. Plasma-treated PTFE samples were glued with epoxy adhesive, and the peel strength was measured. The adhesion was found to be enhanced significantly. Even for samples treated with pure He plasma, the peel strength increased by a factor of 5.1, and with the addition of 1 vol.% of ammonia gas by a factor of 6.6. Interestingly, the authors attributed increased adhesion to the formation of amine groups despite the marginal concentration of C–N groups compared to O–C=O determined from the XPS high-resolution C1s spectra. A feasible explanation of the observations reported by Li et al. [13] is illustrated in Figure 2. Helium plasma sustained at high pressure (Li et al. [13] reported 5 bar) is a significant source of VUV radiation (Figure 2a), which arises from the resonant relaxation of the He_2_* excimers [16]. The absorption depth of such low-wavelength radiation is of the order of 10 nm [17], so the photons cause radiation damage and, thus, the formation of dangling bonds (Figure 2b). Dangling bonds interact chemically with oxygen upon exposure to air to form polar oxygen-containing functional groups (Figure 2c). The addition of ammonia water (or any other molecules) suppresses the radiation from the He_2_* excimers [16]. Still, it causes the dissociation and excitation of radicals (Figure 2d), so the PTFE surface is simultaneously treated with VUV radiation arising from H, H_2_, O, and the radicals (including NH_x_) which interact chemically with the dangling bonds and cause extensive surface functionalization with polar functional groups (Figure 2e).

Antipova et al. [18] probed another fluorine-rich polymer widely used in some industries and for tissue engineering, polyvinylidene fluoride (PVDF). They used helium plasma at atmospheric pressure and at a discharge power of 9 W for the treatment of the PVDF samples in order to increase their wettability. Unlike Li et al. [13], Antipova et al. found a significant increase in surface roughness after the plasma treatment. AFM imaging taken on the surface area of 2.5 µm × 2.5 µm revealed the increased roughness from about 2.5 to 15 nm after treating the samples for 90 s. Intriguing, the WCA dropped from 82° to only 59° and 53° after 60 s and 90 s treatments. They characterized the samples by X-ray diffraction and concluded that plasma treatment does not induce any structural modifications, as the positions and relative intensities of the diffraction peaks remained identical for untreated and plasma-treated samples. Antipova et al., however, found a significant modification in Young’s modulus. While it was about 450 MPa for untreated samples, it dropped to 70 and 40 MPa for samples treated for 60 and 90 s, respectively. The interaction of helium plasma with PVDF is similar to that illustrated in Figure 2a–c, except that the relatively low discharge power did not assure extensive bond breakage by absorption of VUV radiation. The authors attributed such a significant reduction in the substrate surface elasticity to chemical etching induced by the He plasma, which disrupted the cross-linking in the polymer matrix. The authors used plasma-treated samples to study the adhesion and viability of stem cells. They found a significant increase in the density of adhered cells in samples treated for 90 s, while the differences between untreated samples and samples treated for 60 s were marginal. On the other hand, cell viability increased by 10 and 12% for samples treated for 60 and 90 s, respectively. The main improvement was observed qualitatively by examining cells with an optical microscope. While cells were overstressed when grown on untreated PVDF, they were adopted well on plasma-treated substrates and formed aggregates.

**Figure 3:**
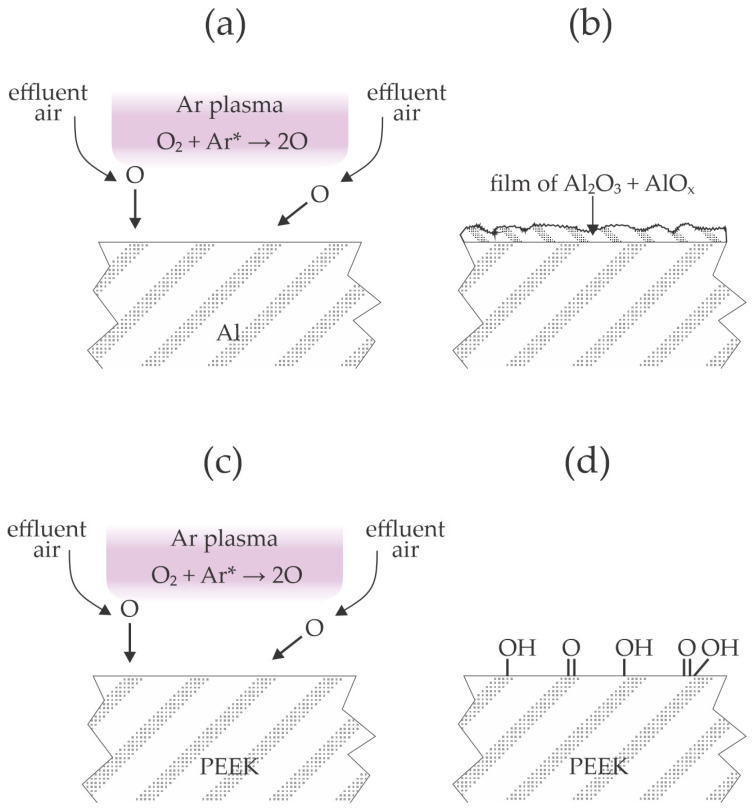
Interaction of 60 MHz atmospheric pressure argon plasma with aluminum (**a**,**b**) and PEEK (**c**,**d**).

Plasma polymer treatment has helped increase the tensile shear strength between aluminum and polymer sheets after hot pressing at 320 °C. Takenaka et al. [19] used atmospheric pressure plasma sustained in argon with an RF generator that operated at the frequency of 60 MHz. The plasma plum extended outside of the quartz tube, so admixing of the effluent air most likely occurred. Polyether ether ketone (PEEK) was selected as the polymer, and the aluminum strip was made from an A1050 grade alloy (containing at least 99.5% Al). The tensile shear strength for untreated materials was about 7 MPa. When the aluminum strip was treated with plasma and the polymer was untreated, the shear strength was as low as 1 MPa, so the treatment of the Al materials with plasma worsened their adhesion capability with the polymer upon hot pressing. When both aluminum and PEEK strips were treated, the shear strength increased to about 9.5 MPa. The best results, however, were reported when only the polymer strip was treated by plasma because the shear strength, in this case, was as large as 13 MPa. This value is higher than when the authors used an adhesive to join both materials. Cohesive and interfacial delamination were reported when the PEEK was treated by plasma and aluminum was untreated. The beneficial results were reported only for the prolonged plasma treatment of PEEK because the shear strength stabilized only after 5 min of plasma treatment. Before, the shear stress was pretty linear with the plasma treatment time. The results Takenaka et al. [17] reported are illustrated in Figure 3. The selected discharge frequency causes significant heating of the gaseous plasma at high pressure. Oxygen from the effluent air dissociates in the jet of Ar plasma (Figure 3a), and the O atoms cause the formation of a relatively thick and rough oxide film on the aluminum surface (Figure 3b). The authors showed scanning electron microscope (SEM) micrographs and EDX and XPS results, which firmly confirmed the formation of the rough oxide film on the aluminum surface. The adhesion of a PEEK foil on such an oxidized aluminum surface is illustrated in Figure 1b. When only the PEEK sheet was treated by plasma (Figure 3c), the adhesion with an Al strip was optimal because the oxygen atoms formed in plasma caused the formation of polar functional groups on the polymer surface (Figure 3d). After hot pressing, some oxygen from the polymer surface interacted with the aluminum strip to ensure highly improved adhesion. When both materials were treated with plasma, the effect of polar groups was marginal since the aluminum had already been oxidized by plasma treatment.

Kosmachev et al. [20] studied the influence of plasma treatment on the interlayer sheer strength of laminates consisting of carbon fiber tapes and interlayers of polyphenylene sulfide (PPS), which served as a thermoplastic binder. The surface of the carbon fiber tapes was treated with pulsed discharges sustained in the air. The maximum voltage of the power supply was 56 kV, and the pulse duration of 10 ns was selected. The treatment times were varied between 5 and 20 min. XPS revealed 23 at.% oxygen even for untreated carbon fibers, and it increased to 33 at.% after 15 min of plasma treatment. High-resolution XPS C1s peaks revealed almost three times larger concentrations of the carbonyl groups after plasma treatment, but the concentration of C–O bonds remained relatively intact. The laminates were prepared in a mold heated to 340 °C at a pressure of 6.5 MPa. The interlaminar shear stress was measured systematically versus the plasma treatment time of the carbon fibers, and the ultimate values were 37, 41, 47, 50, and 52 MPa for untreated samples and samples treated for 5, 10, 15, and 20 min, respectively. The relative increase in the shear stress at the optimal conditions was 40%. SEM micrographs of the cleaved surfaces revealed that the failure occurred strictly along the binder–fiber interface for untreated samples, whereas for those treated with plasma for 15 min, some adhered fragments of the fractured binder were evident on the surface of the carbon fibers.

The same team also probed the adhesion between PEEK polymers and carbon fiber tapes. In the paper [21], the authors studied the mechanical properties of laminates using 0.25 mm thick PEEK foils as thermoplastic binders. The discharge parameters were the same as in [20], and so were the selected treatment times. SEM micrographs showed the localized etching of carbon fibers after prolonged plasma treatments. The temperature during hot pressing was 400 °C, the pressure was 6.5 MPa, and the pressing time was 30 min. The ultimate shear strength increased with increasing plasma treatment time and assumed 48, 52, 68, 73, and 72 MPa for untreated samples and samples treated for 5, 10, 15, and 20 min, respectively. The uniaxial tensile tests (in the direction of reinforcement) were also reported in [21]. Interestingly, the uniaxial tensile strength for samples treated with plasma for 15 min was 20% lower than for laminates synthesized without plasma treatment of the carbon fibers. The team [21] did not probe plasma treatment of PEEK, which was found beneficial by Takenaka et al. [19].

**Figure 4:**
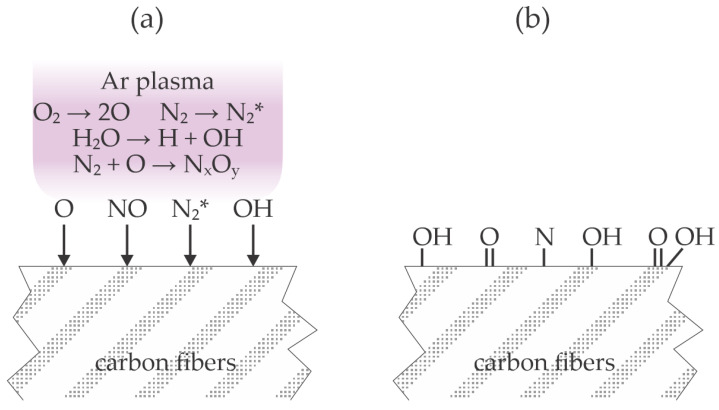
Interaction of air plasma with carbon fibers (**a**) and resulting surface finish (**b**).

The effect of plasma treatment in the configuration adopted by Kosmachev et al. is illustrated in Figure 4. Ambient air contains significant concentrations of water vapor and carbon dioxide. All molecules are partially dissociated, preferentially those with the lowest dissociation energy (O_2_, H_2_O, CO_2_), and nitrogen is excited to metastable electronic states and also vibrational states (Figure 4a). The excited nitrogen molecules are likely to form nitric oxides. The nitric oxides and radicals (predominantly O, OH, and H) interact chemically with the carbon fibers and form polar surface functional groups (Figure 4b).

Jung et al. [22] treated a carbon fiber-reinforced thermoplastic polymer with atmospheric pressure plasma sustained in nitrogen by a dielectric barrier discharge operating at the frequency of 13.56 MHz and voltage of 13.5 kV. The treatment time was 2 min. No wettability measurements were performed, but high-resolution XPS C1s spectra revealed a significant increase in the concentration of the hydroxyl and carboxyl bonds. Intriguingly, no nitrogen bond was observed after treatment with a plasma sustained in nitrogen. The polymer substrate was then coated with an epoxy-based adhesive, covered with an aluminum strip, pressed, and cured. The lap shear stress was about 10 MPa for untreated polymer substrates and 22 MPa for plasma-treated ones. An even larger shear stress of 27 MPa was observed when the plasma-treated carbon fiber-reinforced thermoplastic polymer was covered with a monolayer of mercapto silane. The improved adhesion was explained by the chemical interaction between the adhesive and oxygen-containing functional groups on the polymer surface. The large concentration of oxygen and no nitrogen on the polymer surface after the plasma treatment indicates that the effluent ambient air caused quenching of the nitrogen molecular metastables, similar to the experimental setup used by Kosmachev et al. [20], who also found a minimal concentration of nitrogen on the polymer surface after treatment with air plasma, so the treatment adopted by Jung et al. [22] is illustrated in Figure 4.

Kim et al. [23] treated polypropylene (PP) strips with air plasma sustained at atmospheric pressure and a discharge power as large as 1000 W. The distance between the plasma nozzle and the polypropylene samples was 1 cm, and the transverse speed was 0.25 m/s. The WCA of the untreated samples was about 87° and dropped to about 61° after the plasma treatment. Plasma treatment did not cause a significant modification of the PP morphology, and XPS showed an increase in the oxygen concentration from 7 to 12 at.%. The high-energy tail of the high-resolution XPS C1s spectra showed significant oxidation of the polypropylene sample even before plasma treatment. The authors found the cleavage of C–C bonds in the PP by radicals generated during plasma treatment. The artificial leather made from polyurethane (PU) fabrics was pressed onto the PP samples using PU-based water adhesive as a glue. The sample temperature during pressing at the pressure of 5 bar was 65 °C. There was no adhesion between the PP substrate and the artificial leather before the plasma treatment. After plasma treatment, however, the force needed to separate the two materials with a width of 25 mm was as large as 50 N. The adhesion strength was about the same as in the parallel test using commercial primers for pre-treatment of the PP substrates. The nitrogen concentration on the PP surface after the plasma treatment was just above the detection limit of the XPS instrument, i.e., 0.9 at.%. The surface effects upon plasma treatment, as adopted by Kim et al. [23], are illustrated in Figure 4.

Some authors reported only a marginal increase in the adhesion after plasma treatment of the substrates. Ondiek et al. [24] studied the influence of plasma treatment on the adhesion properties of wood–polypropylene composites. The wood fibers were pre-coated with PP, and the fibers, PP granules, and a compatibilizer (maleic anhydride graft polypropylene) were compounded in a kneading device, pulverized, and finally molded at 200 °C in order to obtain specimens for mechanical tests according to the JIS K7139-A32 standard [25]. The composites were treated with gaseous plasma. No details about the plasma or discharge parameters were disclosed, but the treatment reduced the WCA from 108 to 25°. The composite was, therefore, highly hydrophilic, taking into account some other reports about the hydrophilization kinetics of polypropylene [23]. Different concentrations of wood fibers were added to the composite, and acrylic resin was used as a binding material. The ultimate tensile strength increased from 35 to 35.8 MPa for neat polypropylene, 40.5 to 41.1 MPa for the composite with 25% wood fibers, and 50.7 to 52.4 MPa for the composite with 25% wood fibers.

### 3.2. Low-Pressure Plasmas

Low-pressure plasmas have been used for treating solid materials on the industrial scale for decades [7]. The main advantage of low-pressure plasmas over atmospheric pressure is a much lower collision frequency and a negligible frequency for three-body collisions, which is as low as 1 Hz at the pressure of 100 Pa. Indeed, the loss of molecular radicals in the gas phase is marginal, so the dissociation fraction of molecules is large even at low discharge power per plasma volume. The properties of low-pressure plasmas are governed by surface and not gas-phase reactions. The short mean free path at low pressure enables a moderate electron temperature and high kinetic energy of ions impinging on the surface. The kinetic energy of ions bombarding the surface of a treated material can be adjusted by biasing the samples in a broad range up to a few keV. Due to the marginal loss of reactive plasma species in the gas phase, the low-pressure plasmas expand in large volumes, so the uniform plasma is sustained in chambers with a volume of several m^3^ [26].

**Figure 5:**
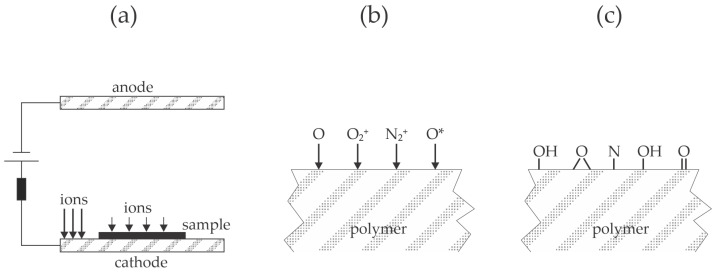
Illustration of the low-pressure DC glow discharge (**a**); radicals and ions from the air plasma sustained by the DC glow discharge (**b**) interact with the polymer surface and cause rapid functionalization (**c**).

Piskarev et al. [27] treated polyethylene naphthalate foils with a low-pressure plasma sustained in the air by a direct current (DC) glow discharge—the discharge operated at 800 V and a current of 50 mA. The foils were placed either on the anode or the cathode. A few seconds of plasma treatment caused increased wettability, and the water contact angle stabilized at about 10° after several 10 s of plasma treatment. Polymer foils treated for 60 s were glued with a solution of polyurethane rubber in acetone and ethyl acetate (PU) or an ethylene vinyl acetate copolymer (EVA). They were heated at 100 °C, and then the peel resistance was determined according to the ASTM 1876-2001 standard [28]. The peel resistance was poor for untreated polyethylene naphthalate foils at 40 and 110 N/m for EVA and PU, respectively. The plasma-treated foils exhibited a much larger peel resistance of about 900 N/m for samples placed on the anode of the DC glow discharge. A significant difference in the adhesion force for samples placed on the cathode was reported. Namely, the force was about 750 N/m for EVA and 400 N/m for PU. The authors found the only difference between the samples treated on the anode and cathode in the roughness evaluated from the measured AFM images, which was 2.1 and 2.5 for samples on the anode and cathode, respectively. Still, another difference is the heating of the electrodes. The DC glow discharge is characterized by the large voltage drop in the cathode sheath between the bulk plasma and the cathode surface [29]. Positively charged ions move randomly in the bulk plasma but are accelerated within the sheath, where they bombard the cathode with a high kinetic energy corresponding to the discharge voltage, so heating is unavoidable. The positive ions suffer some collisions within the sheath since the mean free path of gaseous particles at 15 Pa is roughly 1 mm. Positive ions thus bombard the polymer edge with moderate kinetic energy, but the rest of the polymer foil is at floating potential, so the kinetic energy of ions bombarding the polymer is only about 10 eV, as illustrated in Figure 5a. On the other side, the anode is not heated significantly due to the minimal voltage drop across the anode sheath. The dissociation fraction of oxygen molecules in the air plasma sustained at 15 Pa is considerable (Figure 5b), which explains the quick functionalization of the polymer surface with polar oxygen functional groups and, thus, appropriate wettability, as illustrated in Figure 5c.

**Figure 6:**
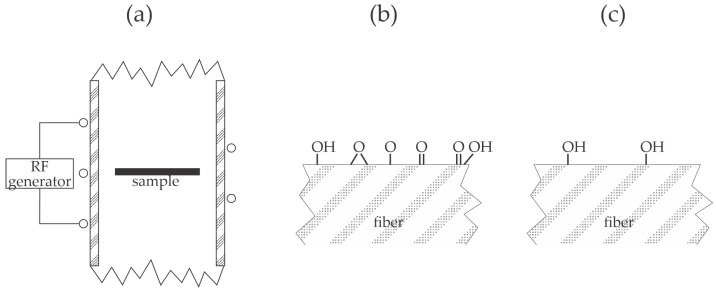
Illustration of inductively coupled plasma (**a**), the surface finish at low discharge power (**b**), and at high discharge power (**c**).

Liu et al. [30] treated 5 µm carbon fibers with a low-pressure inductively coupled radio frequency (RF) plasma in the E-mode sustained in the air at the pressure of 30 Pa. The discharge power varied between 100 and 400 W. The high-resolution XPS C1 spectra revealed that the composition of the carbon fibers was similar to that of the polyamides. The high-resolution C1s spectra of the plasma-treated samples at 100 W caused the appearance of O–C=O groups at a concentration as large as 11%. Further treatment caused a decrease in the intensity of the O–C=O group to 10, 9, and 2% for discharge powers of 200, 300, and 400 W, respectively. No apparent correlation between the concentration of other groups and the discharge power was evident from the C1s spectra. The interlaminar shear strength of composites prepared from the fibers and polyimide resin was 61 MPa for untreated samples and increased to 64 and 68 MPa for 100 and 200 W discharge powers, respectively. However, the shear strength decreased with a further increase of the discharge power and assumed 63 and 62 MPa at 300 and 400 W, respectively. The decrease in the shear strength at elevated powers could be explained either by heating the fibers during plasma treatment and thus fast hydrophobic recovery or the formation of loosely bonded low molecular weight fragments at higher powers. Specifically, AFM showed a significant increase in the roughness: 110 nm for untreated samples and 130, 210, and 330 nm for samples treated at 100, 200, and 400 W, respectively. A peculiarity of electrodeless discharges sustained in glass tubes is a considerable dissociation fraction of molecules, particularly oxygen [31]. As mentioned earlier, the surface reactions are always exothermic, so samples with small dimensions like fibers of diameter 5 µm quickly heat to elevated temperatures. Highly polar functional groups are unstable at elevated temperatures [32], so the etching of polymer materials prevails over functionalization at large discharge powers. Adequate surface functionalization and adhesion of a coating is thus achieved only at a relatively small discharge power. Liu et al. [30] chose a treatment time as long as 900 s, so the fibers of small diameter heated to prohibitively high temperatures, especially at large discharge powers. The thermal effects explain the low oxygen concentration on the fiber surface and the loss of adhesion forces at large power. The effect is illustrated in Figure 6.

**Figure 7:**
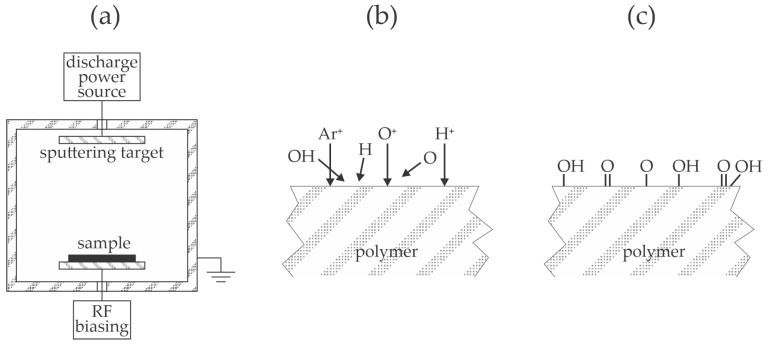
The schematic of the device useful for polymer activation and deposition of a thin metal film (**a**), interaction of plasma species with polymer surfaces (**b**), and resulting surface finish at moderate treatment times (**c**).

Wang et al. [33] studied the adhesion of copper layers on polyimide and PEEK resins and proposed an illustration of the bonds responsible for better adhesion on plasma-treated polymers. Various pre-treatments by chemical methods and sandblasting were used before the plasma treatment. The interlayer between the polymer substrate and the copper film was a thin titanium layer deposited by magnetron sputtering. The polymers were first treated with argon plasma at the pressure of 0.2 Pa and biased against the plasma potential. The treatment times were 10, 30, 80, and 150 s. After activating the polymer surfaces with argon plasma, the samples were coated first with titanium and then copper using sputter deposition without breaking vacuum conditions. No apparent correlation between the roughness on the nanometer scale and tensile strength was found for polyimide substrates, but the authors reported an excellent correlation for PEEK—a larger surface roughness resulted in a higher tensile strength, as illustrated in Figure 1a. Many polymer samples were treated with Ar plasma at the fixed bias of −100 V. For the polyimide substrates, the tensile strength at −100 V did not depend on plasma treatment time since it was always around 1 MPa. On the other hand, PEEK samples exhibited tensile strengths of 4, 7, 15, and 6 MPa for samples treated for 10, 30, 80, and 150 s, respectively. The thermal effect explains the drop in the tensile strength for the longest treatment. As mentioned earlier, biasing causes the acceleration of positively charged ions, bombarding the polymer surface with significant kinetic energy. The authors selected pressure as low as 0.2 Pa, where the mean free path is well below 1 cm, so the sheath next to the polymer sample is collisionless—at biasing to −100 V, the kinetic energy of positively charged ions bombarding the polymer surface is 100 eV. Sandblasting before plasma treatment caused a significant increase in the tensile strength for polyimide samples at 4.2, 5.8, 5.7, and 6.2 MPa for samples treated for 10, 30, 80, and 150 s, respectively. On the contrary, the sandblasting of PEEK substrates before plasma treatment caused an insignificant increase in the tensile strength; it was 8, 10, 8, and 11 MPa for samples treated for 10, 30, 80, and 150 s, respectively. The influence of the bias voltage was examined as well. The tensile strength decreased monotonously with the absolute value of the bias voltage for polyimide, while for PEEK, it peaked at the bias voltage of −100 V. The experimental system adopted by Wang et al. [33] is illustrated in Figure 7a. The residual atmosphere in hermetically tight low-pressure systems always consists of water vapor, which fully dissociates in Ar plasma, so the polymer sample is subjected to neutral and positively charged OH, O, and H radicals (Figure 7b). The velocity of the charged particles is perpendicular to the polymer surface, while neutral radicals move randomly in the gas phase. The radicals cause surface functionalization (Figure 7c) and improved wettability, but prolonged treatment time results in a high polymer temperature, which explains the loss of adhesion at the longest treatment time.

**Figure 8:**
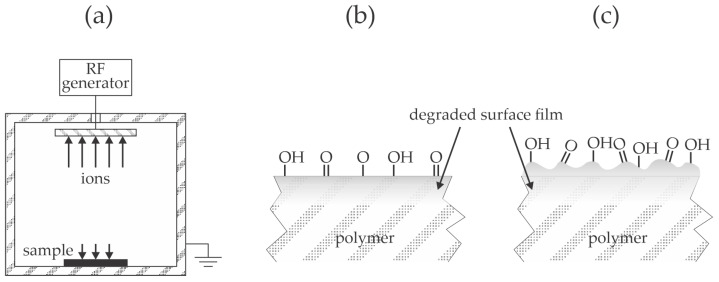
Illustration of capacitively coupled discharge for treatment of polymers with plasma (**a**) and the surface finish after a short treatment of several 10 s (**b**) and treatment for 120 s (**c**).

Wu et al. [34] used a low-pressure plasma sustained in the air by a capacitively coupled RF discharge operating at the frequency of about 50 kHz and a nominal power of 200 W to modify the surface of PP films. Plasma treatment caused a gradual increase in the wettability with the water contact angles of 107, 67, 64, 62, and 8° after plasma treatment for 0, 15, 30, 60, and 120 s, respectively. No explanation for a dramatic decrease in the WCA between 60 and 120 s of plasma treatment was provided, but it could be explained by the degradation of the polymer surface film due to the bombardment with moderately energetic plasma ions. A relatively thick film of polyurethane adhesive of 5 mm was deposited on the PP substrates, and the lap shear stress was measured according to the ASTM D3136 standard [35]. The shear stress increased monotonously with plasma treatment time and correlated well with wettability. The maximal load was 35, 64, 75, 80, and 110 N/cm^2^ for samples treated for 0, 15, 30, 60, and 120 s, respectively. The improved adhesion was stable because, after a week of storage, the maximal load remained practically the same as for freshly synthesized samples. Furthermore, the annealing of samples treated with plasma for 30 s for one day caused a further increased shear strength to 120 N/cm^2^. The authors explained the improved adhesion by chemical interaction between the hydroxyl groups formed on the PP surface during plasma treatment and the isocyanate group from the adhesive to form a urethane bond. The experimental system used by Wu et al. [34] is illustrated in Figure 8a. Low-pressure capacitively coupled discharges are renowned for DC self-biasing of the electrodes [36], and the smaller electrode (powered electrode in Figure 8a) assumes larger biasing. A polymer sample is placed on the grounded electrode. The powered electrode is subjected to extensive bombardment with positively charged ions, while the larger electrode (usually grounded, as shown in Figure 8a) is subjected to moderately energetic ions. As long as the electrically non-conductive samples like polymer foils are thin compared to the sheath thickness, the surface of the treated material assumes the same DC biasing as the backing electrode. The synergy of bombardment by positively charged ions and absorption of VUV radiation modifies the properties of the PP surface film, as illustrated in Figure 8b. The prolonged treatment causes further surface film modification and nano-structuring, as illustrated in Figure 8c. A theoretically spotless description of the modification of the polymer surface film by ion treatment is provided in [37]. The combination of rich surface morphology and functionalization with polar functional groups, as illustrated in Figure 8c, leads to the super-hydrophilic surface finish [38], which explains the WCA as low as 8° at the longest treatment time.

Wu et al. [39] also treated PP films with oxygen plasma for 30 s and studied the adhesion of 2 K polyurethane (PU) adhesion strength. The lap shear adhesion test was performed according to the ASTM D3163 standard method [35]. No details about oxygen plasma were provided, but they likely used the same configuration illustrated in Figure 8a. The adhesion strength increased from 24 for untreated PP films to 29 N/cm^2^ for those treated with oxygen plasma. The authors added amino silane and epoxy silane to the adhesive and reported marginal differences in the adhesion strength.

## 4. Correlations and Paradoxes

**Figure 9:**
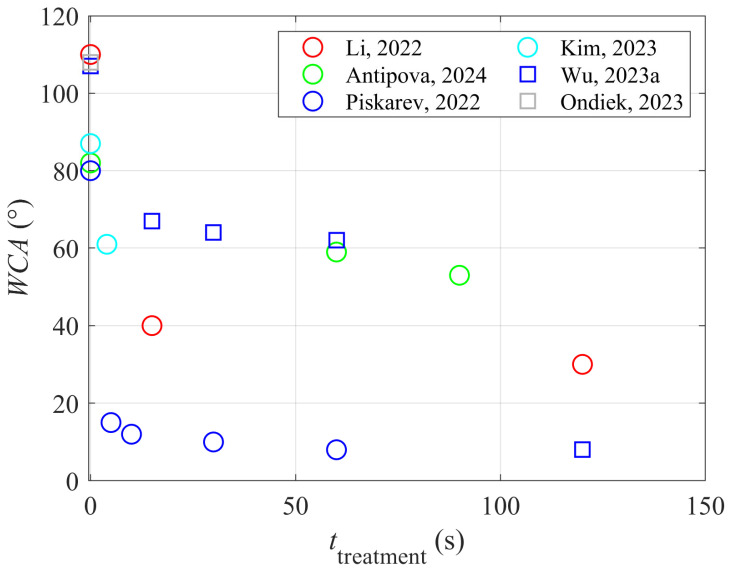
Water droplet contact angle versus the plasma treatment time [13,18,23,24,27,34].

The authors cited in Section 3 used different experimental systems and have not reported plasma parameters (i.e., the fluxes and doses of plasma species, including the positively charged ions, neutral reactive species, and energetic photons), so the reported results seem incomparable. Still, some general correlations could be drawn based on results reported by different authors for different polymer materials. Most authors reported the plasma treatment time. Figure 9 represents a plot of the water droplet contact angle (WCA) versus the treatment time. All probed polymers were moderately hydrophobic, with the WCA for untreated samples between 80 and 110°, and all became hydrophilic after the plasma treatment. The decrease in the WCA with increasing treatment time varies for different polymers, but the trend is evident: longer treatment times enable better wettability. The WCA remains moderate in some cases, but some authors reported an almost super-hydrophilic surface finish (WCA below 10°). As mentioned earlier and explained in detail elsewhere [38], the super-hydrophilic surface finish of polymers is only achieved if the surface is rough on the sub-micrometer scale and functionalized with polar groups, as illustrated in Figure 8c.

**Figure 10:**
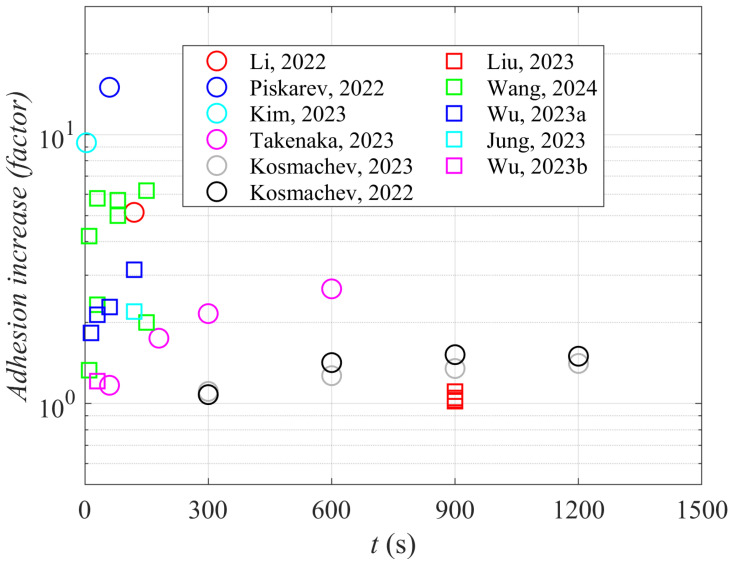
The ratio between adhesion after and before the plasma treatment (adhesion increase) versus the treatment time [13,19,20,21,22,23,27,30,33,34,39].

**Figure 11:**
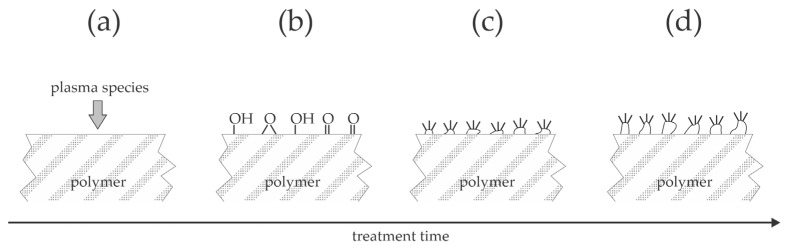
The formation of agglomerates of molecular fragments on the polymer surface. (**a**) The polymer is exposed to plasma species, which cause functionalization (**b**). Moderate doses of plasma species (prolonged treatment time and/or rather large discharge power) cause the formation of well functionalized molecular fragments (**c**), which may agglomerate after large doses of plasma species (**d**).

**Figure 12:**
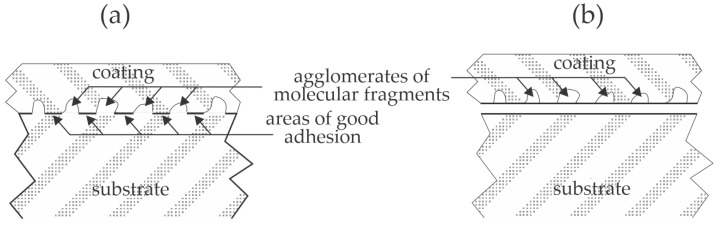
Illustration of the effect of molecular fragment agglomerates on the adhesion. (**a**) The coating wets the substrates well, so the coating fills all gaps on the surface, but the area of good adhesion is limited to the surface free from agglomerates. (**b**) The agglomerates are peeled from the polymer surface after the adhesion test, so the shear strength is inadequate.

Longer plasma treatment times do not necessarily ensure better adhesion. Figure 10 is a plot of the adhesion increase versus the treatment time. The adhesion increase is defined as the shear strength after the plasma treatment divided by the shear strength of untreated samples. The reported results are scattered and are not sound with the evolution of WCA (Figure 9). Namely, the higher wettability should enable better interaction of the joined materials because it suppresses the effect illustrated in Figure 1b. The paradox is explained by loosely bonded molecular fragments formed during prolonged treatment with gaseous plasma. As mentioned above, plasma treatment always causes breaking bonds between atoms in the polymer’s surface film. All authors who probed the surface composition by XPS reported a significant increase in the oxygen concentration in the surface film probed by this technique, whose probing depth is several nm. Oxygen may replace hydrogen to form hydroxyl or epoxy bonds, which is supposed to be the first effect of the plasma treatment [10,40,41], but they also break bonds in the polymer chain [42] and trigger the degradation of polymer material [43]. The degradation leads to etching, i.e., the release of volatile molecules, but incomplete oxidation leads to the formation of molecular fragments that remain on the polymer surface [44]. Large agglomerates of such fragments were reported for plasma-treated PEEK and polyether sulfone [45,46]. These molecular fragments are loosely bonded to the polymer surface and can be removed even by gentle rinsing with water [47], although the surface of these fragments is well oxidized. Thus, the well oxidized fragments of the original polymer substrate contribute to improved wettability; they are weakly bonded to the surface to the extent that they are easily removed during adhesion tests. The formation of the molecular fragments is likely to result from the synergy of VUV radiation, positively charged ions, and perhaps also neutral radicals, and is yet to be elaborated. The evolution of the molecular fragments is illustrated in Figure 11, and their influence on the peeling test is illustrated in Figure 12. The appearance of the fragments was not reported in the articles analyzed in Section 3 of this paper, so it is impossible to quantify their influence on the adhesion properties.

## 5. Recommendations and Conclusions

In this study, recent papers on the adhesion of plasma-treated polymers were reviewed. Some authors reported the details about the experimental setup used to treat polymer material, but most only mentioned the type of discharge used to sustain the gaseous plasma, the gas pressure, the treatment time, and the increase in adhesion. Such an incomplete description of the experimental conditions does not enable the reproducibility of treatments at different laboratories. The authors are recommended to provide details about the experimental setups because the plasma parameters depend significantly on the dimensions and geometry of the plasma reactor, the coupling of the power supply, the biasing of the polymers against the plasma potential, and so on.

Even if the details mentioned above are provided, the plasma parameters are still challenging to deduce, so plasma characterization is recommended. A simple introduction to diagnostic methods for plasmas useful for polymer treatments is provided in [15]. Non-equilibrium gaseous plasma sustained in reactive or noble gases with an admixture of reactive gas is rich in neutral radicals, such as neutral atoms in the ground state. The atom density and, thus, the flux of atoms onto the polymer surface, depends enormously on the peculiarities of the discharges used for sustaining non-equilibrium gaseous plasma [31]. However, the typical dissociation fraction is often around 10% in plasmas with the neutral gas translational temperature close to the room temperature [7] as long as the plasma is sustained in a chamber made from dielectrics. The dissociation fraction is significantly smaller in metallic chambers [48]. The appropriate neutral radicals for tailoring the surface wettability of polymers are O atoms and OH radicals [43]. Small doses cause functionalization of the polymer surface without significant etching and degradation of the polymer chains, and the OH radicals favor the formation of hydroxyl groups [49]. The first effect of treatment with O atoms is also the formation of hydroxyl groups [10,41], but the doses exceeding 10^20^ m^−3^ cause the formation of other oxygen-containing functional groups when treating polymers both at atmospheric pressure [50] and in low-pressure reactors [51].

Other reactive species should be considered when interpreting the results of plasma treatment. Plasma is a source of VUV radiation, with the photon energy exceeding the binding energy of atoms in polymer materials. The effect of this radiation has been studied for decades and was also found significant for treating polymers on an industrial scale [52]. As much energy absorbed by gaseous plasma as about 10% was found to be transferred to VUV radiation [53], so it is of crucial importance for the hydrophilization of fluorine-containing polymers [13].

The effect of positively charged ions on the surface wettability of polymers is regarded as marginal when treating the samples with weakly ionized non-equilibrium gaseous plasma, because the ionization fraction is orders of magnitude smaller than the dissociation fraction [7]. Still, if the polymer surface is biased, as explained in [33], the ions play an important role, since their high kinetic energy causes radiation damage to the surface film. Large doses of energetic, positively charged ions will cause excessive heating of the polymers since practically all ions’ energy is spent on the heating of the polymer surface. The elevated surface temperature causes rapid loss of surface wettability [32].

Finally, it is worth stressing again that high hydrophilicity, as indicated by a low water droplet contact angle, does not necessarily result in optimal adhesion. The formation of agglomerates of well-oxidized molecular fragments on the polymer surface explains the paradox. The fragments add to the wettability but are easily removed from the polymer surface, so the adhesion of any coating on a polymer, rich with molecular fragments, will be inadequate.

## Data Availability

This is a review article; therefore, no new data were generated.
